# EASE: Clinical Implementation of Automated Tumor Segmentation and Volume Quantification for Adult Low-Grade Glioma

**DOI:** 10.3389/fmed.2021.738425

**Published:** 2021-10-05

**Authors:** Karin A. van Garderen, Sebastian R. van der Voort, Adriaan Versteeg, Marcel Koek, Andrea Gutierrez, Marcel van Straten, Mart Rentmeester, Stefan Klein, Marion Smits

**Affiliations:** ^1^Department of Radiology and Nuclear Medicine, Erasmus MC, Rotterdam, Netherlands; ^2^Brain Tumor Center, Erasmus MC Cancer Institute, Rotterdam, Netherlands; ^3^Medical Delta, Delft, Netherlands

**Keywords:** brain tumor, low-grade glioma (LGG), segmentation (image processing), magnetic resonance imaging (MRI), clinical translation, lesion quantification

## Abstract

The growth rate of non-enhancing low-grade glioma has prognostic value for both malignant progression and survival, but quantification of growth is difficult due to the irregular shape of the tumor. Volumetric assessment could provide a reliable quantification of tumor growth, but is only feasible if fully automated. Recent advances in automated tumor segmentation have made such a volume quantification possible, and this work describes the clinical implementation of automated volume quantification in an application named EASE: Erasmus Automated SEgmentation. The visual quality control of segmentations by the radiologist is an important step in this process, as errors in the segmentation are still possible. Additionally, to ensure patient safety and quality of care, protocols were established for the usage of volume measurements in clinical diagnosis and for future updates to the algorithm. Upon the introduction of EASE into clinical practice, we evaluated the individual segmentation success rate and impact on diagnosis. In its first 3 months of usage, it was applied to a total of 55 patients, and in 36 of those the radiologist was able to make a volume-based diagnosis using three successful consecutive measurements from EASE. In all cases the volume-based diagnosis was in line with the conventional visual diagnosis. This first cautious introduction of EASE in our clinic is a valuable step in the translation of automatic segmentation methods to clinical practice.

## Introduction

Magnetic resonance (MR) imaging plays a key role in the management of low-grade glioma (LGG) as a method for measuring treatment response and for regular surveillance during periods of watchful waiting. LGG are known to show constant slow growth (1), until—in adults—they inevitably transform to a more malignant type. The early growth rate of the T2-weighted hyperintense region is a known prognostic factor for malignant progression (2) and overall survival (3), so the reliable quantification of growth may be a valuable tool for clinical decision making (4). However, due to the anisotropic growth and irregular size it can be difficult to evaluate slow growth on consecutive imaging using a visual assessment or 2D measurement (5). Volumetric measurements are preferred for the assessment of early growth due to their reproducibility and sensitivity to subtle changes (6), but a manual segmentation would require an effort that is unrealistic in clinical practice.

Automatic segmentation of glioma has shown great advances in recent years due to the release of public datasets and the development of artificial intelligence (7). A recent method described in Kickingereder et al. (8) has been shown to be a reliable alternative for the prognostication of glioma, comparable to the current clinical standard of 2D measurement according to the RANO criteria. Although these criteria apply specifically to high-grade glioma and the measurement of enhancing tumor (6), the performance evaluation in Kickingereder et al. also shows an almost perfect quantification of non-enhancing abnormalities on T2-weighted FLAIR imaging. This makes it potentially suitable for the assessment of volume changes in non-enhancing low-grade glioma.

Due to the clear clinical need of volume quantification in LGG, we decided to implement a segmentation pipeline and integrate it in the existing clinical workflow of the Brain Tumor Center, Erasmus MC Cancer Institute, Rotterdam. This introduced a new measurement tool in the radiologists' toolbox, which we named EASE: Erasmus Automated SEgmentation. With a new tool come potential risks to patient safety and quality of care, which need to be considered in the design of the software and protocols for its use.

For the clinical implementation of this segmentation pipeline, we identified potential risks and practical challenges. The main concern was that of incorrect tumor segmentations resulting in incorrect volume measurements. Further risks were found in software updates over time, potentially leading to unreliable or inconsistent volume measurements, and finally in the incorrect interpretation of volume measurements at time of diagnosis. These risks and the design choices to address these are described in more detail in sections Materials and Equipment and Methods, and an overview is shown in Table 1.

**Table 1:** Overview of identified risks and measures to address those risks.

**Risk**	**Measure**
Segmentation errors	Quality check in annotation interface (section Quality Assessment)
Inconsistencies due to updates	Reference dataset and version control (section Validation and Version Control)
Incorrect interpretation of volumes	Design guidelines for usage (section Diagnosis)
	Storage of segmentations in PACS (section Reporting)

This work describes the design of both the technical implementation of EASE and its integration into the clinical workflow, to ensure quality of results and prevent incorrect interpretation of the resulting volume measurements. Furthermore, an initial evaluation of the software was performed in which both the success rate and clinical impact of the volumetric assessment were measured.

## Materials and Equipment

**Figure 1:**
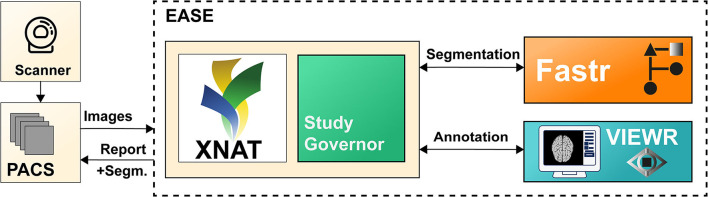
Illustration of the different components of EASE. Images are sent from the PACS and added to the XNAT (9) database. The data and state manager (Study Governor) triggers the processing using Fastr (10). After successful processing, the results can be checked in the VIEWR. A report, including the delineations, is sent back to the PACS.

This section describes the software implementation of EASE. Each scan assessed with EASE goes through a number of processing steps: (1) The images (pre- and post-contrast T1-weighted, T2-weighted, and T2-weighted FLAIR) are received and stored (section Data Management); (2) The segmentation is generated (section Segmentation); (3) The segmentation is checked by a radiologist (section Quality Assessment); (4) A report is generated and sent back to the PACS (section Reporting). A data and state management tool is used to manage the state of each scan and launch processing tasks, in order to balance the workload on the server and enable monitoring of errors in the process. The global software design and data flow are shown in Figure 1. The software components for data management, processing and annotation are all open-source, both as separate components and as an adaptable containerized framework^1^ using Docker (11).

### Data Management

The scan is sent from the PACS (Vue PACS, Carestream Health, v12.2.2.1025) to a dedicated workstation where the scan protocol is automatically checked and the required MR sequences (see section Segmentation) are automatically selected. The images are then stored on a local XNAT database (v1.7) (9), which forms the common database for all further processing steps. The images are stored for a maximum of 6 months to allow for monitoring of the algorithm performance over time, while avoiding unnecessary risk to patient privacy.

### Segmentation

The input for the segmentation consists of four MR sequences: pre- and post-contrast T1-weighted, T2-weighted and T2-weighted FLAIR imaging. The pipeline consists of the following steps: first, the images are converted from DICOM to Nifty images using dcm2niix (v1.0.20171215) (12) and co-registered to the postcontrast T1-weighted scan using Elastix (v4.8) (13). Then, they are skull-stripped using HD-BET (git commit 98339a2) (14) and MR bias fields are corrected using N4ITK (using SimpleITK v2.0.2 for Python) (15). The resulting images are used as input for HD-GLIO (v1.5) (14, 16), producing the final delineation of both the enhancing tumor and non-enhancing hyperintensities on T2-weighted FLAIR. Although bias correction is not included in the recommended preprocessing for HD-GLIO, initial tests showed that this improves the performance of the segmentations for scans from our clinic. This pipeline was found, in initial experiments, to perform well on representative images in our center. The Fastr workflow engine (v3.2) (10) was used to integrate these different tools in a robust pipeline.

### Quality Assessment

Although the underlying segmentation algorithm, HD-GLIO, was evaluated in a large number of scans and found to be reliable (8), an initial evaluation in our center found that our pipeline does not provide perfect segmentations in all scans of low-grade glioma (see section Validation and Version Control). The manual quality assessment of segmentations is therefore essential for the use of EASE in clinical practice. To enable this assessment within a clinical workflow, a dedicated interface was developed for the radiologist to easily assess the segmentation.

The main purpose of the quality assessment is to prevent failed segmentations from being used for a volume-based diagnosis. Additionally, the same quality assessment can be used for the initial validation of the algorithm, prospective evaluation, and continuous monitoring of the segmentation quality. Therefore, besides a binary check on the usability of the segmentation, a more refined quality assessment scoring system was included. Important factors in the design were usability and prevention of human errors.

**Figure 2:**
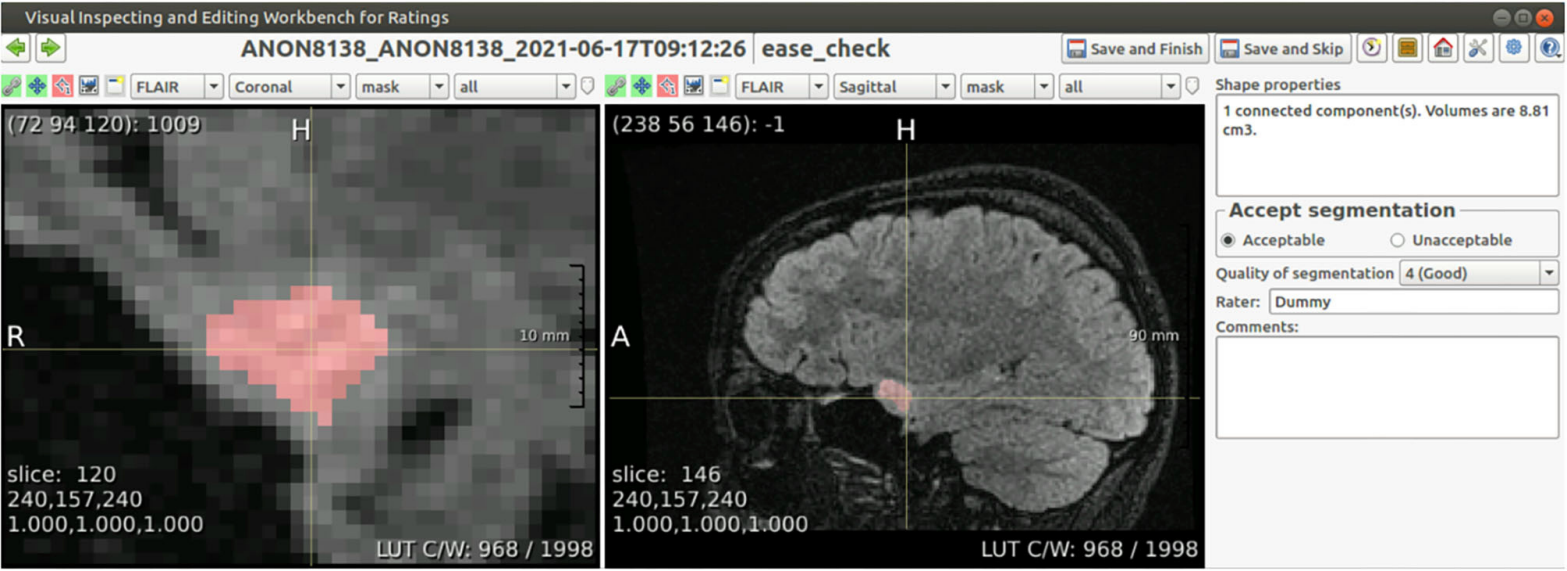
Screenshot of the annotation interface. Both image panels can be controlled to show different scan sequences, imaging planes, to change the contrast, to zoom in or out, or to set the overlay transparency. Besides the required annotation of quality, the panel on the right shows the volume of each connected component in the segmentation, and allows for free text comments that are included in the report.

The interface shows the segmentation as an overlay over all four co-registered scans, and allows for basic interaction through scrolling, manipulation of the contrast, and selecting sequences and imaging planes. The radiologist is asked to evaluate the segmentation both in a binary way (ACCEPTABLE/UNACCEPTABLE) and on an ordinal scale (rating of 1–5, where 5 is the best score). As an additional sanity check, specifically to prevent unnoticed false positives, the interface also lists the number of connected components in the segmentation together with their volumes. Segmentations deemed UNACCEPTABLE cannot be used for diagnosis. A screenshot of the interface is shown in Figure 2.

### Reporting

**Figure 3:**
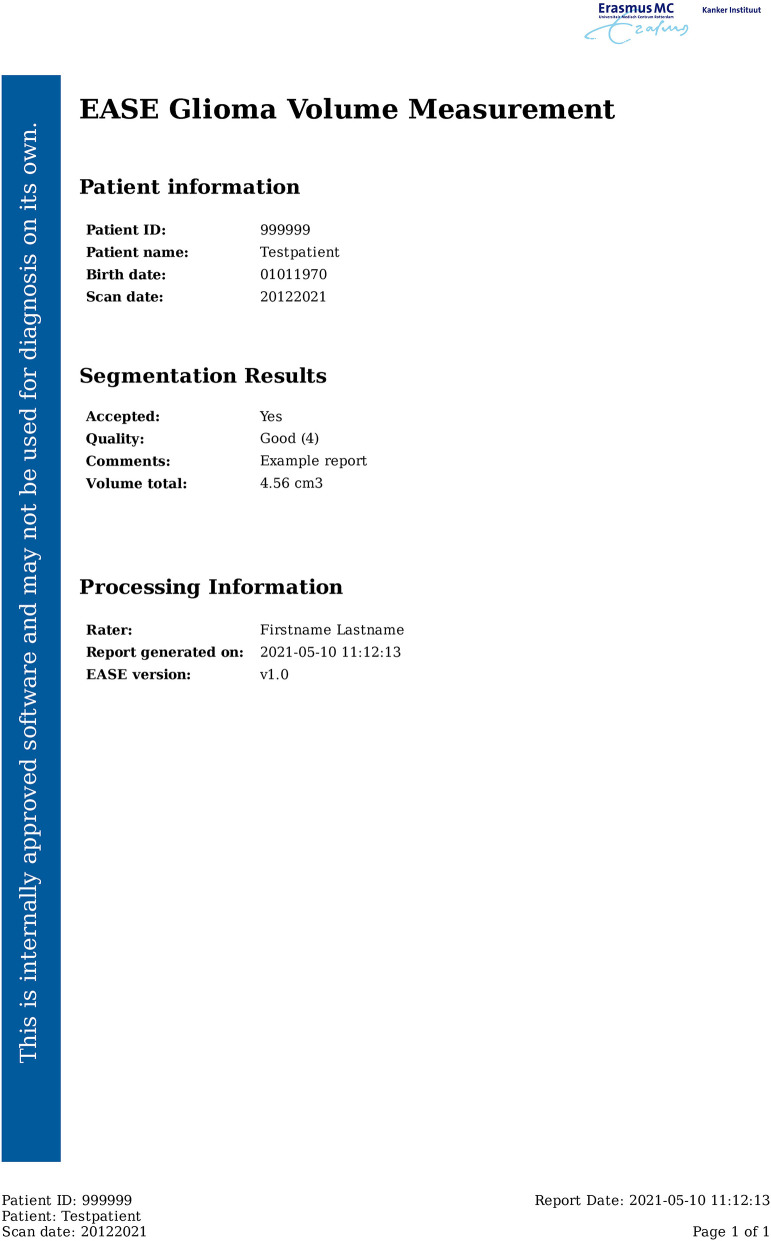
Example of the EASE report.

Results of the EASE assessment are sent back to the PACS in the form of a report (see Figure 3) exported as DICOM file. This report contains the quality assessment, current software version and details of the scan session. Volume measurements are included only if the segmentation is deemed acceptable, to make sure rejected segmentations are not used for diagnosis. In addition to the report, the segmentations are shown as delineations on the T2-weighted FLAIR and post-contrast T1-weighted scan. It would have been possible to store results as a DICOM Structured Report and DICOM SEG respectively, but conventional DICOM images were preferred as not all viewers used in the clinic supported these formats.

## Methods

This section describes the protocols for usage of EASE in diagnosis (section Diagnosis), the measures for software validation and version control (section Validation and Version Control), and the method for initial evaluation in clinical practice (section Evaluation in Clinical Practice).

### Diagnosis

The purpose of volume measurements produced by EASE is to assess therapy response or progression by estimating tumor growth. The standard clinical procedure for estimating growth is to compare the current measurement to two previous measurements and measure the difference in size, with a manual quantitative measurement of two perpendicular diameters if possible, as described in the RANO guidelines (6). The EASE software provides an automated 3D alternative to the existing measurement. However, as the EASE software has not been tested extensively in this setting, we decided that the existing 2D method should still be performed before using EASE. The volume measurements provided by EASE can lead to further insight and even a different diagnosis, but if there is a discrepancy between the two assessment methods leading to a different conclusion, the diagnosis should be made in consensus with a second radiologist.

**Figure 4:**
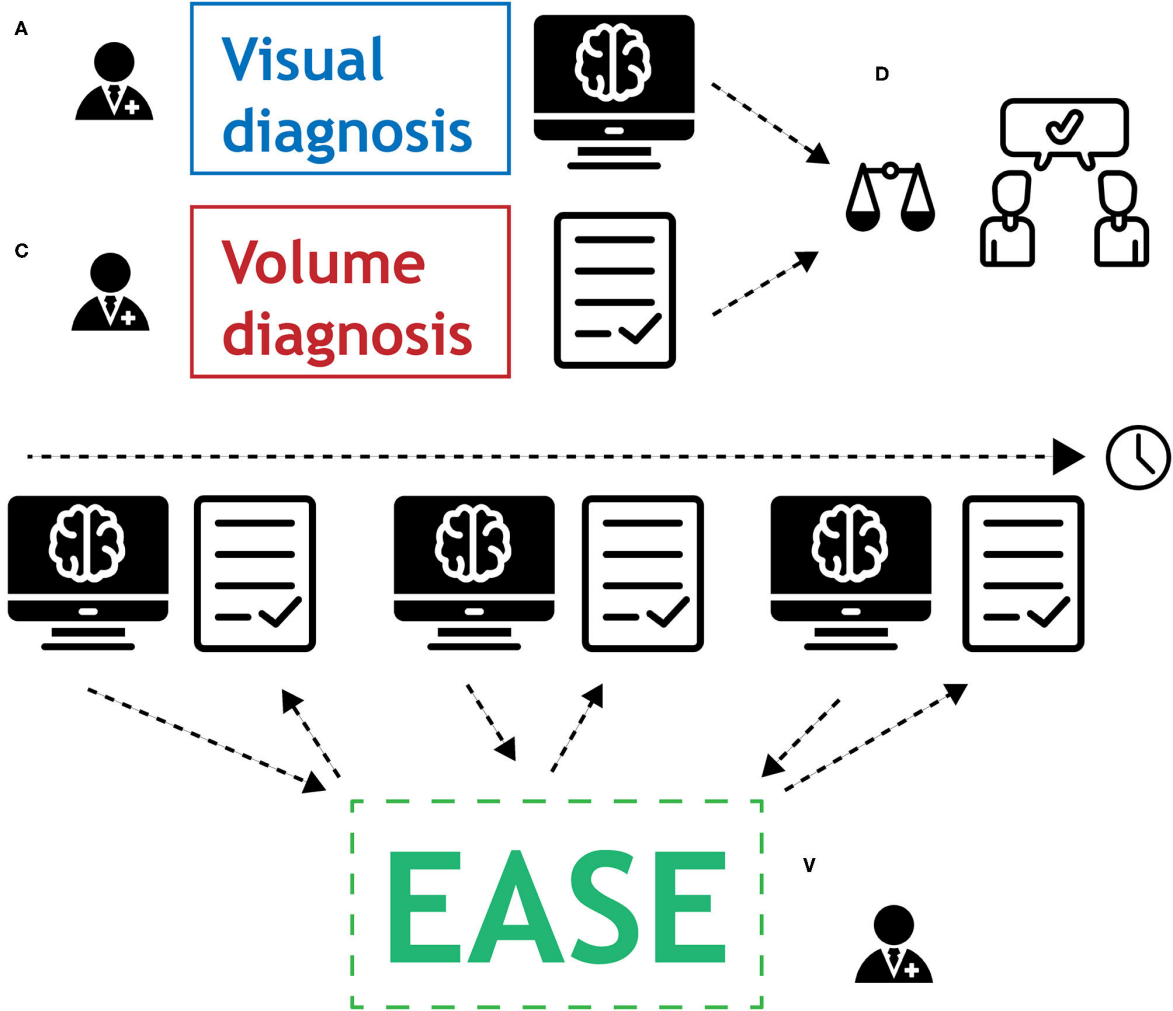
Graphical representation of the protocol for the use of EASE in clinical practice. **(A)** Radiologist applies conventional method for visual diagnosis. **(B)** Segmentations are produced by EASE and assessed separately. **(C)** Radiologist interprets volume measurements. **(D)** If volume measurements lead to change in diagnosis, a second radiologist is consulted for a consensus conclusion.

The following protocol is in place for the interpretation of automatic volume measurements in clinical practice. The complete workflow is illustrated in Figure 4.

1. Two prior reference scans are selected for the assessment (in addition to the current scan).
2. The radiologist assesses the scan using the routine 2D RANO measurement.
3. EASE is applied to all three scans and the segmentations are checked for quality and acceptance. If any scan was already processed and checked previously, this does not have to be repeated.
4. If any of the segmentations are rejected, a volumetric assessment is not possible.
5. If all segmentations are accepted, the volumes can be compared.
6. If the volume measurements lead to a change in interpretation compared to the initial assessment after step 2, a second radiologist must be consulted. This second rater first forms an independent opinion of the diagnosis. If this is in line with the first radiologist's opinion, this finalizes the conclusion. If not, both radiologists discuss together how their findings are best described in the report, clearly indicating the uncertainty regarding the findings.

The radiological report clearly describes how each assessment is done (2D RANO, 3D EASE) and how the conclusion is reached. If there was a discrepancy between the two methods, leading to a consensus diagnosis, this should be reflected in the report.

### Validation and Version Control

Before deploying the EASE workflow/pipeline, and after any subsequent update, the segmentation quality should be tested in a reference dataset that is representative of the target domain. For this purpose, 20 scans were selected of patients with non-enhancing LGG. All sessions were surveillance scans of patients who had undergone surgical resection, but no further treatment, of LGG. For these scans, the same quality assessment as described in section Quality Assessment was performed by an experienced neuroradiologist.

It is essential that updates to the software do not cause a bias in volume that might skew the diagnosis. Therefore, a protocol for software updates was established that allows updates of the processing pipeline while ensuring the continued quality and consistency of the volume measurements. The protocol is as follows:

1. In case of an update, the reference dataset of 20 segmentations is processed again with EASE.
2. The segmentation results are compared to earlier versions of the software. If there is no change in the segmentation, the update can be deployed.
3. If there is a change in results, the manual validation is repeated with the new results.
4. If the qualitative scores are equal or improved with respect to the previous version, the update can be deployed.
5. If the update causes substantial differences in volume (defined as a difference >25%) in any of the accepted segmentations in the reference dataset, the new version is considered incompatible with previous versions and volume results cannot be compared between versions. A warning is included in subsequent EASE reports, so that radiologists know when they have to re-assess previously segmented reference scans with the updated version of EASE.

### Evaluation in Clinical Practice

To evaluate the impact of automated segmentation and volume quantification, an observational study was performed for 3 months from first introduction of the software in the clinic. The study protocol was reviewed and approved by the internal review board (MEC-2021-0530). Users were asked to complete a survey after each patient in whom EASE was applied, to measure the success rate of EASE in practice and the rate at which volume quantification leads to a change in diagnosis.

To assess the treatment response or tumor progression in non-enhancing LGG three consecutive volume measurements are required, as the standard clinical procedure is to compare the current scan to two former scans. Therefore, patients were excluded if EASE was applied to the first scan after surgery. Furthermore, patients were excluded if any contrast enhancement was found, which would automatically lead to a diagnosis of tumor progression irrespective of volume measurements.

For each of the included patients, the radiologist was first asked whether EASE had led to a successful diagnosis. Although the success rate of a single segmentation can be extracted from the quality assessments made in the user interface, the success of a full diagnosis requires three accepted segmentations from the same patient. If the diagnosis was unsuccessful, the user was asked to submit the reason for failure.

**Figure 5:**
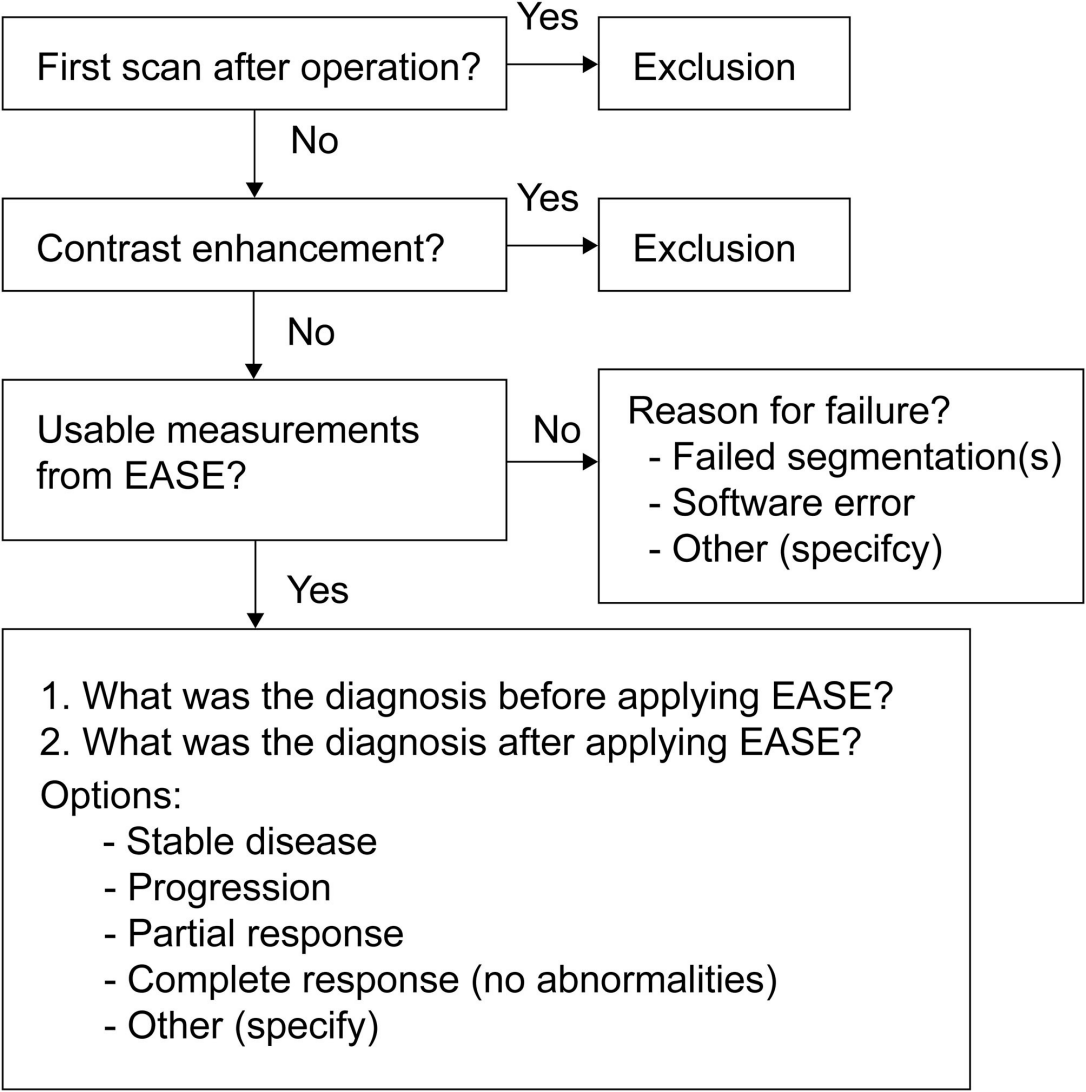
Flowchart of the survey on the usage of EASE in clinical practice.

When the volumetric diagnosis was successful, the radiologist was asked to categorize both the visual (2D) diagnosis and the volume-based diagnosis (through EASE) as progression (PD), stable disease (SD) or treatment response. These results, combined with the quality assessments made in EASE for the individual scans, were used to measure the success rate of EASE and the impact on the clinical diagnosis. The full user survey is shown in Figure 5 in the form of a flowchart.

Additionally, for the purpose of a quantitative comparison, measurements were made according to the 2D RANO-LGG guidelines (6) if at all possible, measuring two perpendicular diameters of the lesion. As these lesions are often irregular in shape, the diameters were measured in the portion of the lesion that could be measured most reliably.

## Results

Of the 20 scans in the reference set, which were processed and evaluated before deployment of EASE, 13 (65%) were considered acceptable for clinical volume measurement. The quality scores are summarized in Table 2.

**Table 2:** Results of reference dataset of 20 MR scans at initial release of EASE.

**Acceptance**		
	ACCEPTABLE	13 (65%)
	UNACCEPTABLE	7 (35%)
**Quality**		
	Perfect (5)	0 (0%)
	Good (4)	10 (50%)
	Fair (3)	6 (30%)
	Poor (2)	2 (10%)
	Terrible (1)	2 (10%)

*Scans were annotated for acceptability and quality by an experienced neuroradiologist*.

EASE was released for local use in Erasmus MC on 25 May 2021, and the evaluation in clinical practice was performed from 1 June 2021 until 19 August 2021.

During the evaluation period, 55 patients were included in the clinical evaluation, meaning that their visual diagnosis was performed and a volume-based diagnosis was attempted. The patient characteristics are summarized in Table 3. A successful diagnosis requires three consecutive scans per patient, and in total 162 scans were segmented by EASE and checked by a radiologist. In one of the patients, the two reference scans were not submitted to EASE after the first segmentation was already rejected and in another scan the segmentation failed due to a software error.

**Table 3:** Characteristics of 55 patients included in the evaluation of EASE in clinical practice.

**Patient characteristics: (total)**	55
**Age (years)**	
Median (minimum–maximum)	54 (26–76)
**Sex**	
Female	24
Male	31
**Tumor type**	
Oligodendroglioma	19
Astrocytoma	25
Oligo-astrocytoma	2
Presumed low-grade glioma (no tissue diagnosis)	9
**Time after surgery (months)**	
Median (minimum–maximum)	80 (5–307)
**Time after last treatment (months)**	
Median (minimum–maximum)	67 (5–307)
**Treatment**	
Radiotherapy	33
Chemotherapy	33
Surgical resection	41
**Time between scans from current scan (months)**	
vs. first reference scan, median (minimum–maximum)	14 (7–32)
vs. second reference scan, median (minimum–maximum)	7 (3–20)
**Tumor volume found in successful volume-based diagnosis (mL)**	
Median (minimum–maximum)	13.2 (1.3–77.1)
Oligodendroglioma, median (minimum–maximum)	18.3 (2.1–77.1)
Astrocytoma, median (minimum–maximum)	16.1 (2.1–60.0)
Oligo-astrocytoma	27.8 (9.5–46.1)
Presumed low-grade glioma, median (minimum–maximum)	2.0 (1.3–14.9)

**Figure 6:**
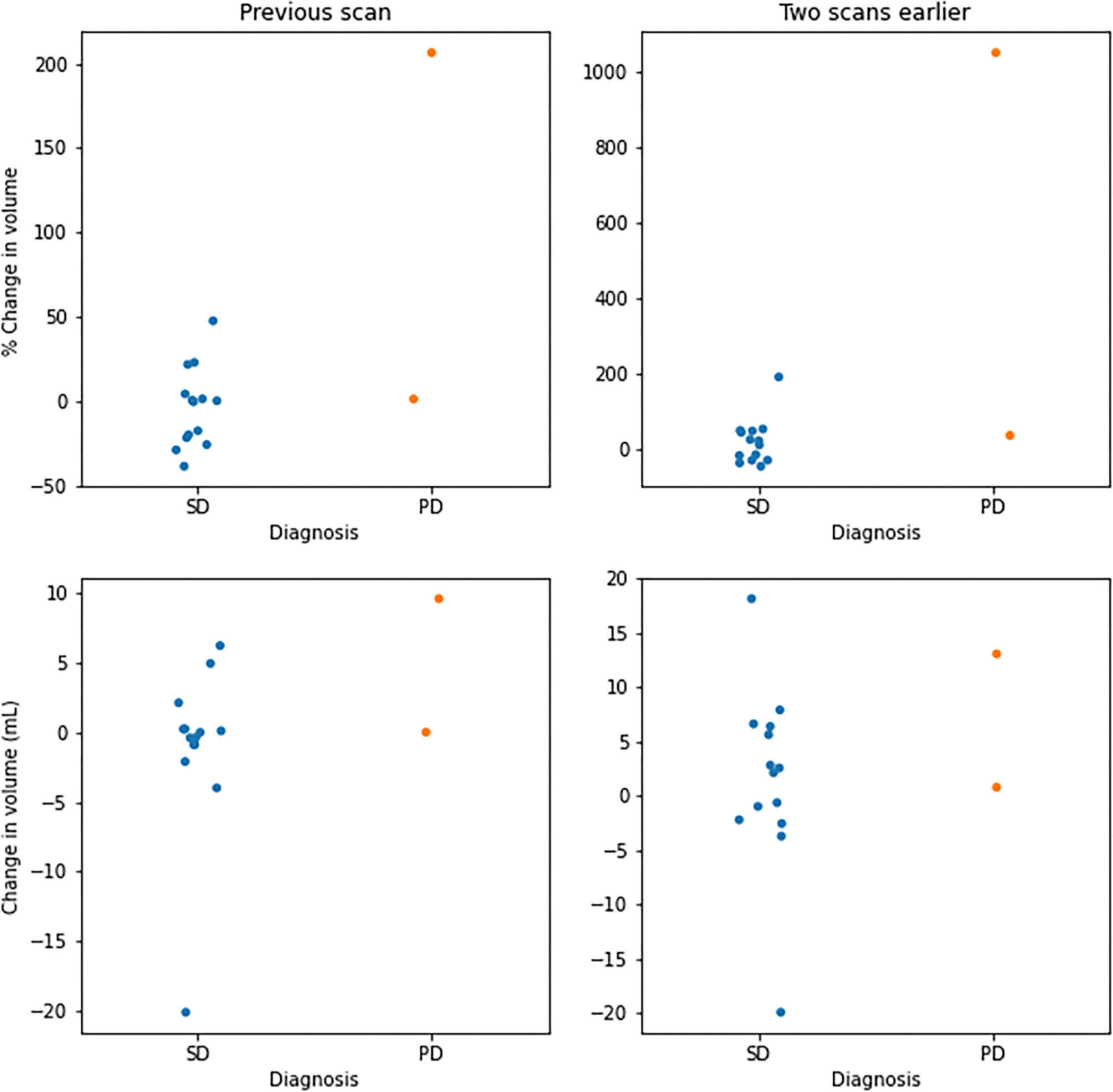
Overview of volume changes in successful volume-based diagnosis. Changes per patient with respect to the previous scan (left) and two scans earlier (right). Values are given in percentage change (top) and change in volume (bottom), separated by diagnoses categorized as stable disease (SD) and progressive disease (PD).

**Figure 7:**
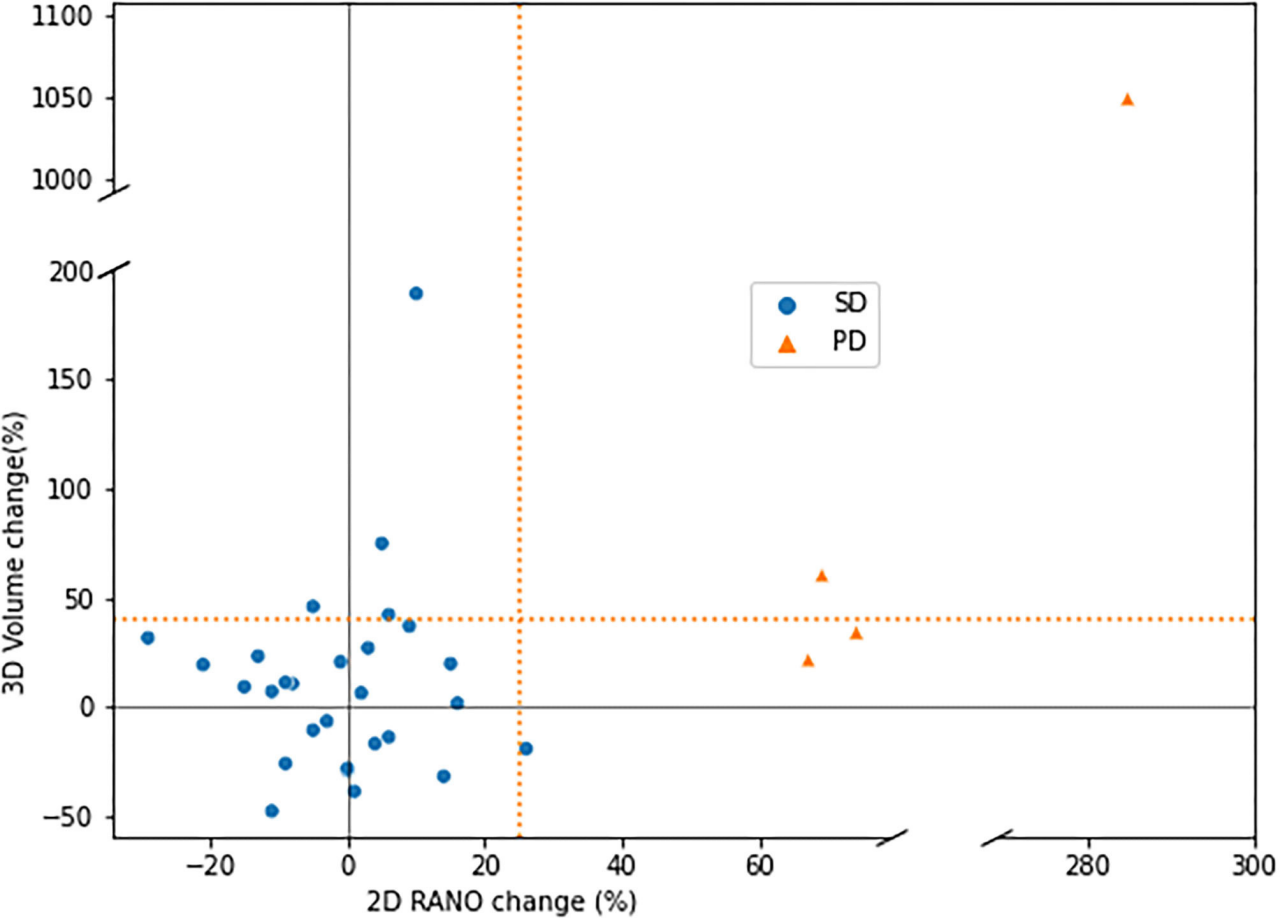
Comparison of measurements using EASE (volume) and 2D RANO (product of two diameters), in percentage change with respect to the first (*t*−2) scan, for patients where both measurements were successful (33 patients). Dotted lines indicate the recommended thresholds for diagnosis of PD.

Of the 162 segmentations generated by EASE, 124 (77%) were accepted by the radiologist. The distribution of quality scores can be found in Table 4. A successful volume-based diagnosis was reached in 36 out of 55 patients. Results of the questionnaire are summarized in Table 5. In all patients where volume-based diagnosis was successful, the volume-based diagnosis made by the radiologist was the same as the conventional visual diagnosis, even though in some cases there was a discrepancy between 2D and 3D measurements as shown in **Figure 7**. Figure 6 shows an overview of the volume differences detected by EASE, separated by diagnosis (stable disease vs. progression). Figure 7 shows a comparison to the 2D RANO measurements for those patients in whom both measurements were possible. Three patients are not included in this figure because the lesion was too small to measure according to RANO guidelines. In four patients, EASE measurements indicated a volume increase of more than 40% while the final diagnosis was SD. These differences in volume could be explained by inconsistencies between the segmentations, possibly caused by differences in intensities on T2-FLAIR, and therefore the radiologist maintained the original visual diagnosis of SD. There were no other reported reasons for considering volumetric measurements longitudinally unreliable.

**Table 4:** Results of annotations entered in EASE in clinical practice.

**Acceptance**		
	ACCEPTABLE	124 (77%)
	UNACCEPTABLE	38 (23%)
**Quality**		
	Perfect (5)	15 (9%)
	Good (4)	87 (54%)
	Fair (3)	33 (20%)
	Poor (2)	17 (10%)
	Terrible (1)	10 (6%)

*During the first 3 months of usage, 162 scans were annotated for acceptability, and quality by five different radiologists*.

**Table 5:** Results of evaluation of EASE in clinical practice.

**Total number of patients assessed**	55
Volume-based diagnosis same as visual diagnosis	36
- Stable disease	32
- Progression	4
Volume-based diagnosis different from visual diagnosis	0
No usable results	19
- Segmentation unacceptable	17
- Inconsistent segmentations	1
- Software error	1

*Radiologists were asked to fill in a questionnaire after assessing a patient with EASE, which requires the successful segmentation of three consecutive scans*.

**Figure 8:**
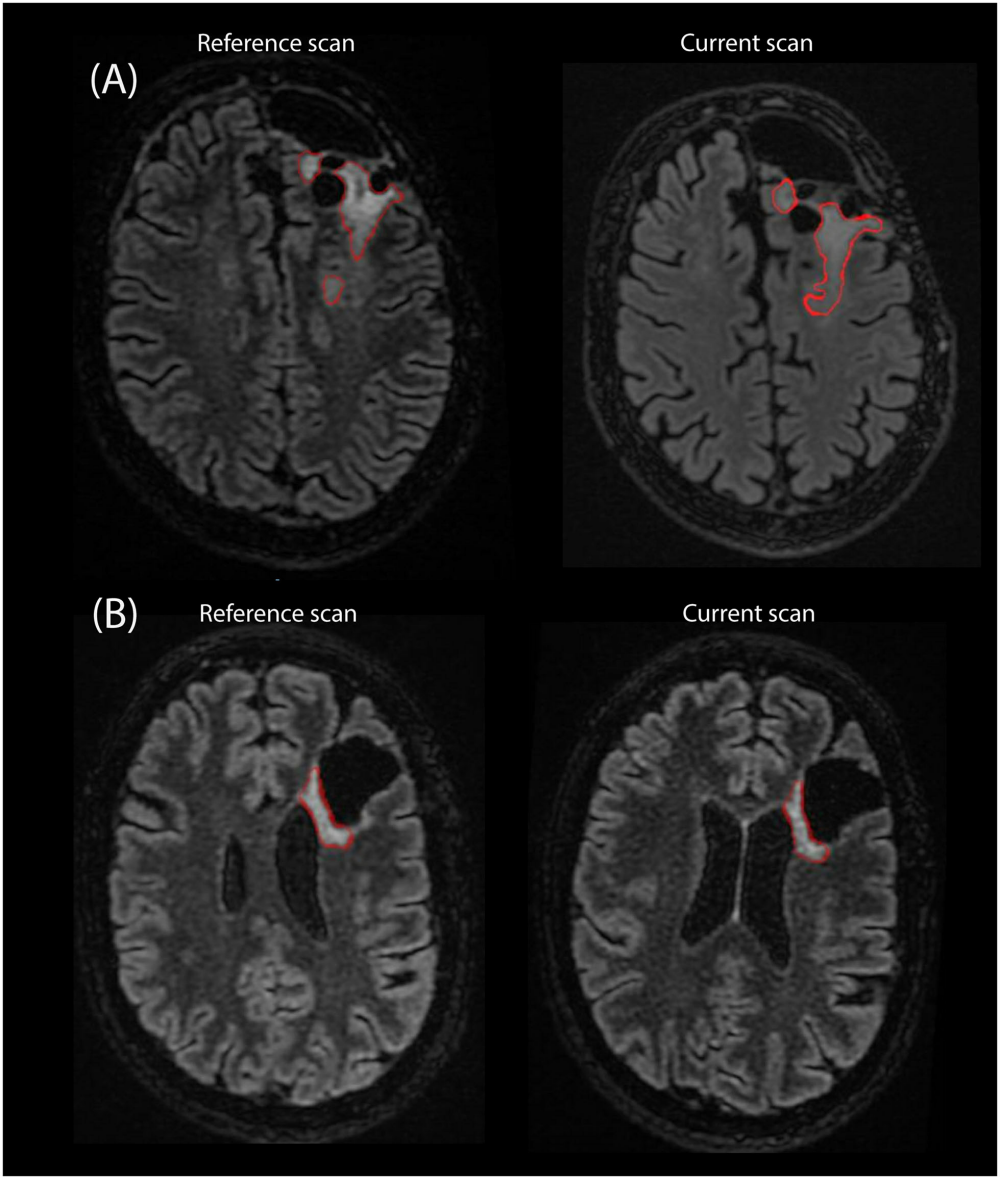
Example of segmentations as they are stored in PACS as an overlay on the T2-FLAIR scan from two consecutive timepoints. **(A)** Two consecutive scans of patient where EASE segmentations were considered inconsistent by the radiologist. **(B)** Two consecutive scans where a volume-based diagnosis of stable disease could be made.

Of the failed cases, 19 could be attributed to the rejection of one of the segmentations and two failed diagnoses were attributed to a different reason. Specifically, in one case a segmentation was missing due to a software error, and in another case all segmentations were accepted by the radiologist but the final volume results were considered unusable due to inconsistencies between the segmentations across the three timepoints. Figure 8 shows examples of segmentations made by EASE: two consecutive delineations that were considered inconsistent and two consecutive delineations from a successful volume-based diagnosis.

## Discussion

A clinical segmentation pipeline ‘EASE’ was implemented to perform automated 3D volume measurements in LGG. As the effect of such a measurement on clinical decision making is still unknown, and perfect performance of the algorithm cannot be expected, several steps were taken to ensure patient safety and monitor results.

The main purpose of this work is to establish the protocols and tools to allow the first introduction of a new, potentially valuable diagnostic tool into clinical practice. From the initial reference dataset, with 7 out of 20 segmentations rejected, it is clear that the quality assessment remains an essential step in the usage of EASE. First results from clinical practice indicate a similar success rate of 74% for individual scans, and approximately half of the patients could be successfully diagnosed with three consecutive volume measurements. However, since the sample size is limited, with almost exclusively diagnoses of stable disease, so further validation of the performance is required to draw firm conclusions on the expected success rate. It must be noted that the segmentation of non-enhancing LGG is particularly difficult due to their diffuse border and varying signal intensity on particularly T2-FLAIR imaging. Furthermore, the underlying deep learning solution, HD-GLIO, was evaluated mostly on high-grade glioma. The current application is therefore aimed at a different, and possibly more challenging patient group and while our results show that a clinical application is feasible, but a more reliable segmentation is needed to facilitate efficient diagnosis.

The results confirm that automatic segmentation of low-grade glioma during follow-up is not a solved problem, and therefore highlight the importance of the quality assurance protocols and manual checks that are presented in this work, and which are ideally part of any introduction of new assessment tools into clinical practice. EASE facilitates a quantitative measurement of lesions that are often impossible to measure accurately even in 2D, due to their irregular shape, and therefore serves a long-standing wish from the neuro-oncological community to move to a potentially more accurate 3D measurement. In this light, a successful diagnosis in over half of the patients is already a valuable step forward.

The initial evaluation in clinical practice provides valuable feedback on the use of automatic segmentation in low-grade glioma. Notably, it shows that an automatic segmentation method is no guarantee for consistent results. Even though the inter-rater variation is removed through automation, the diffuse border of low-grade glioma can still cause ambiguity in the segmentation. Ideally, an automatic segmentation method would be consistent in its choice of where to set the border, but results from EASE show that slight variations in image intensities between consecutive scans can lead to longitudinal inconsistencies. This means that a critical assessment by the radiologist is still needed even if all segmentations are checked and accepted on an individual basis. In EASE, this is ensured by a workflow that can be easily applied in the clinical routine and the protocol for clinical decision-making described in section Diagnosis. Future technical improvements in the automatic segmentation of LGG should focus not only on improving the quality of individual segmentations, but also on longitudinal stability. For this, assessing the reproducibility of the entire process from scan to measurement would be of value, although this would require repeated measurements within a close enough timeframe to assume no change in tumor volume. Such a set-up is not consistent with clinical practice and would require a dedicated study with funding for additional scanning procedures and full consideration of whether the burden this incurs on patients is justified reproducibility of the entire process from scan to measurement.

This work describes a first and careful implementation of automatic segmentation of LGG in clinical practice. Although the results leave room for improvement for the segmentation method, it is already being applied successfully in approximately half of the patients. In all patients diagnosed thus far, the volume measurements confirm the conventional visual diagnosis, as would be expected, but the volume quantification increases confidence in the diagnosis. Essentially, results show that radiologists are cautious in their use of the measurements. The fact that the segmentations are verified and stored for future reference not only decreases the risk of a false diagnosis, but also increases the confidence of the radiologist when using such deep learning solutions in their clinical practice.

Only four patients were included with a diagnosis of progressive disease (PD), which can be attributed to the fact that the most common sign of PD is the presence of contrast enhancement. This is often accompanied by concurrent volume increase, but these cases were excluded from the study in order to address the diagnostic uncertainty regarding non-enhancing lesions. When comparing the volume change between patients with SD and PD, there is no clear threshold to separate the two categories. Although the RANO guidelines recommend a threshold of 25% change for 2D measurements, which would correspond to a 40% change in volume, the final interpretation is left to the discretion of the radiologist and may depend on other factors, such as baseline volume, the presence or absence of treatment-related white matter abnormalities and the consistency of segmentations longitudinally.

When looking at the 2D RANO measurements there is a clear distinction between SD and PD, even though these measurements do not capture the full extent of the irregular shape and diffuse infiltration of these lesions. From these results it seems that the existing visual diagnosis is still being used as the primary tool to determine tumor growth, but are too few patients showing progression in either method to draw a firm conclusion. Also, it must be noted that these results were gathered in the first months after EASE was released for clinical use.

EASE was put into service prior to the date of application of EU regulation 2017/745 on medical devices (MDR). We are aware that in case of substantial changes in the design or intended purpose of EASE, the requirements of this regulation are applicable. Our approach to ensure quality of results and prevent incorrect interpretation is already in line with the general aim of the MDR.

We think this implementation provides a potential benefit to both the clinicians and researchers, as radiologist receive a valuable tool for the quantification of glioma volume, even if not fully perfected, while researchers receive valuable feedback from clinical practice. In its current form, EASE does not allow for correction of failed segmentations through manual intervention of the radiologist, as this is not feasible in clinical practice. However, the feedback from clinical practice could enable further improvement in the segmentation, whether that is in the preprocessing or by improving the HD-GLIO model in a transfer learning approach, while the clearly defined protocol for software updates ensures patient safety during such future improvements.

## Data Availability Statement

The raw data supporting the conclusions of this article will be made available by the authors, without undue reservation.

## Ethics Statement

The studies involving human participants were reviewed and approved by MEC-2021-0530 (Erasmus MC Rotterdam). Written informed consent for participation was not required for this study in accordance with the national legislation and the institutional requirements.

## Author Contributions

The manuscript was written by KG and reviewed by SvdV, MSm, MSt, and SK. Software development was done by KvG, AV, AG, and SvdV. The protocol for use of the software in the clinic was designed by MSm, MSt, and MR. The evaluation in clinical practice was conceptualized by MSm and KvG and implemented by KvG. All authors contributed to the article and approved the submitted version.

## Funding

KvG was funded by the Dutch Cancer society (project number 11026, GLASS-NL) and the Dutch Medical Delta.

## Conflict of Interest

The authors declare that the research was conducted in the absence of any commercial or financial relationships that could be construed as a potential conflict of interest.

## Publisher's Note

All claims expressed in this article are solely those of the authors and do not necessarily represent those of their affiliated organizations, or those of the publisher, the editors and the reviewers. Any product that may be evaluated in this article, or claim that may be made by its manufacturer, is not guaranteed or endorsed by the publisher.

## Footnotes

^1^https://gitlab.com/radiology/infrastructure/medical-imaging-demo.
